# Dental Procedures in Primary Health Care of the Brazilian National Health System

**DOI:** 10.3390/ijerph14121480

**Published:** 2017-12-01

**Authors:** Suellen R. Mendes, Renata C. Martins, Antônio T. G. Matta-Machado, Grazielle C. M. Mattos, Jennifer E. Gallagher, Mauro H. N. G. Abreu

**Affiliations:** 1 Graduate Programme in Dentistry, Faculty of Dentistry, Universidade Federal de Minas Gerais, Belo Horizonte 31270-901, Brazil; srmendesodonto@gmail.com; 2 Department of Community and Preventive Dentistry, Faculty of Dentistry, Universidade Federal de Minas Gerais, Belo Horizonte 31270-901, Brazil; r.c.martins@uol.com.br; 3 Department of Preventive and Social Medicine, Faculty of Medicine, Universidade Federal de Minas Gerais, Belo Horizonte 30130-100, Brazil; thomaz@nescon.medicina.ufmg.br; 4 Division of Population and Patient Health, Kings College London Dental Institute, Denmark Hill Campus, London SE5 9RS, UK; gueziabh@yahoo.com.br (G.C.M.M.); jenny.gallagher@kcl.ac.uk (J.E.G.)

**Keywords:** primary health care, dental care, health services

## Abstract

The aim of this study was to examine the procedures of primary dental health care performed by oral health teams (OHTs) adhering to the second cycle of the ‘National Programme for Improving Access and Quality of Primary Care’ (PMAQ-AB) in Brazil. A cross-sectional descriptive analysis was performed, across 23 dental procedures comprising preventive, restorative/prosthetic, surgical, endodontic and oral cancer monitoring. Descriptive analysis shows that most of the oral health teams carry out basic dental procedures. However, most of the time, they do not keep adequate records of suspected cases of oral cancer, diagnosis tests or follow-ups, and do not perform dental prosthetic procedures. Data also showed disparities in the average number of procedures performed in each Brazilian geographical region in 2013–2014, ranging from 13.9 in the northern to 16.5 in the southern and south-eastern regions, reinforcing the great social disparities between them. Brazilian regions with the highest volume of dental need deliver the lowest number of dental procedures. The need to tackle inequalities and further shape the supply of appropriate primary health care (PHC) is evident.

## 1. Introduction

The inclusion of oral health teams (OHTs) in the Brazilian National Health System (SUS in Portuguese) and subsequent creation of the National Oral Health Policy, represented the enhancement of primary health care (PHC) and the expansion of dental health-care access to the Brazilian population [1,2,3].

There are currently 24,053 OHTs throughout Brazil [4]. Moreover, there has been a structural improvement in the dental facilities in PHC units, better qualification of OHT professionals (each OHT is composed of one dentist and at least one dental assistant—an oral health auxiliary and/or oral health technician), the provision of dental prostheses in PHC and the expansion of secondary dental health care [3].

This great expansion of access brought with it the need to evaluate the quality of service provided to the population. In this sense, in 2011 the Ministry of Health (MofH) launched the ‘National Programme for Improving Access and Quality of Primary Care’ (PMAQ-AB) to increase access to and improve the quality of services provided by PHC by technically and economically supporting PHC teams. Each team underwent an accreditation process based on the results of an external evaluation and on the analysis of health indicators.

The first cycle was conducted between 2011 and 2012, and evaluated 12,404 OHTs. It was determined that most OHTs performed preventive, restorative and surgical procedures; however, procedures concerning oral cancer and dental prosthetic procedures were performed less frequently [5,6]. The low frequency of performance of these procedures is worrying, especially considering that the Brazilian health system is universal and that adults and the elderly often require such procedures. The second cycle occurred between 2013 and 2014. In contrast to the first cycle, when only 50% of OHTs were able to adhere to the PMAQ; in this second cycle, all primary health-care teams, including OHTs, were included in the evaluation. In addition, the external evaluation instrument was improved in the quantity of variables related to structure, work process and outcomes [5,6]. Serial health assessments are important in verifying whether there have been changes in the performance of health services. However, the scientific literature is rather scarce on this topic.

Thus, the present study aims to describe the procedures of primary dental health care performed oral health teams adhering to the second cycle of the ‘National Programme for Improving Access and Quality of Primary Care’ in Brazil.

## 2. Materials and Methods

This is a cross-sectional descriptive study using secondary data related to the primary dental care procedures performed by oral health team (OHT) adherents to the second cycle of Programme for Improving Access and Quality of Primary Care’ (PMAQ-AB). This survey was based on the classical quality of care framework by Donabedian, in which quality is evaluated using structure, process, and outcome parameters [7]. In the present study, we used data obtained in the external evaluation phase, which involves an interview with the dentists about the work process of the OHTs and verification of documents in the primary health care (PHC) units.

The Brazilian National Health System is a universal health system that allows access to oral care for all age groups, from early childhood to seniors, without direct costs to the population There is a national protocol that sets out a range of basic oral health-care procedures (preventive, restorative/prosthetic, surgical, endodontic, and monitoring of oral health conditions) considered necessary for the epidemiological need of the Brazilian population [1,2,3].

For this phase, an observational instrument was created through a Ministry of Health (MofH) partnership with six teaching and research institutions in Brazil. This instrument was applied by a trained team of health professionals, all of whom had completed university degrees. Questions (mostly of the dichotomous yes/no type) and observations were recorded on tablets using a computer programme specifically designed for PMAQ-AB [5]. After the evaluation cycle, MofH organized the database and made it available to the teaching and research institutions.

In this study we considered the performance reports of 23 basic dental health-care procedures, including preventive, restorative/prosthetic, surgical, endodontic, and cancer monitoring procedures.

Each OHT received one point for each dental procedure performed. As such, each OHT’s score was the sum of the number of dental health procedures, ranging from zero to 23. Therefore, descriptive analysis was performed, showing the proportion of OHTs that performed each dental procedure at the time, in addition to the average number of procedures performed by the teams in the five Brazilian geographical regions (north, north-east, mid-west, south and south-east). Confidence intervals were not calculated because this was a census of OHTs that adhered to PMAQ-AB.

The study was submitted to and approved by the National Ethics Research Council and by the Research Ethics Committee of the Federal University of Minas Gerais (protocol number 02396512.8.0000.5149). We analysed a public and anonymous database from the Brazilian MofH; therefore, it was not necessary to ask for individual informed consent.

## 3. Results

In the second cycle of PMAQ-AB, which occurred between 2013 and 2014, 19,946 OHTs were assessed. Of these, 1832 (9.2%) were disqualified by the PMAQ-AB evaluation criteria because they did not follow the programme recommendations, such as an adequate oral health surveillance system and the presence of the dentist and dental equipment in the PHC unit, resulting in a sample of 18,114 OHTs.

Table 1 shows the frequency of 23 primary dental care procedures performed by the OHTs, grouped by categories of procedures, including preventive, restorative/prosthetic, surgical, endodontic, and cancer monitoring procedures.

Table 2 shows that OHTs performed, on average, 15.5 dental procedures (SD 2.8; range 1–23 procedures). When the average numbers of procedures performed by dentists in the five Brazilian geographical regions were evaluated, it was observed that OHTs from the south and south-east (the more developed regions, accordingly to Human Development Index - HDI) performed a higher number of primary dental procedures, whereas teams from the north (one of the two least developed region, accordingly to HDI) performed, on average, fewer procedures. Teams from the mid-west and north-east regions carried out an average of 15 procedures, which is closer to the Brazilian average.

## 4. Discussion

Descriptive analysis shows that the majority of OHTs carry out a range of basic dental procedures, including individual preventive, restorative, endodontic, and surgical procedures. Although many teams report referral of suspected/confirmed cases of oral cancer, few teams keep adequate records of these. Concerning dental prostheses, half of the teams identify the need, but few in fact perform impressions, cementation and consultation for dental prosthesis evaluation.

Although there have been numerous methodological changes and a large increase in the number of OHTs evaluated between the first and second cycle of PMAQ-AB, the findings agree with the study by Reis et al. [6], who evaluated data from the first cycle of the PMAQ-AB. In general, OHTs perform basic oral health procedures, but do not perform the more complex procedures incorporated in the PHC from the National Oral Health Policy of 2003. This may be due to the lack of preparation of professionals, or lack of technical/structural support for performing such procedures. In addition, it is important to highlight that, even in cases of great skill of the primary care dentists, some procedures require specialized attention, demanding referral of the patient to secondary care [9]. Bias in self-reported data is a limitation of this study and its impact in the information of dental procedures should be checked in future research.

Before the insertion of the OHTs into SUS, access to dental health care was restricted to defined population groups and based on basic preventive care and surgical intervention (oral surgery and restorative procedures) [1]. The creation of a National Oral Health Policy based on the PHC principles of the Alma–Ata Declaration increased the access, infrastructure and training of professionals [10]. However, few OHTs expanded the variety of dental procedures offered to the population, either due to lack of preparation of the dentists or, in some cases, to lack of technical support for performing such procedures, which goes against the needs of the population and against the universality precepts of the SUS.

Although a reduction of edentulism in the Brazilian population was identified by some researchers, tooth loss is still characterized as a relevant public health problem, especially amongst elderly people [11,12]. Dental prosthetic procedures, such as temporary removable prostheses, removable partial prostheses and dentures, are part of PHC, but OHTs are not obligated to conduct this procedure, even though it is encouraged by management and laboratory support is made available. An inadequate frequency of performance of such procedures is observed, given the great need presented by the population for both partial prostheses and crown and fixed bridges. Recently, dental implants were included in secondary dental care in the Brazilian National Health System [1,2,3,6]. It is interesting to observe that impression-taking frequency is lower than that of prosthesis cementation. It is probably that the dental prosthesis cementation procedure is also being performed for patients whose prostheses were not performed under the Brazilian National Health System. This procedure could also include re-cementation of dental prostheses.

Although the majority of OHTs report having a place to which they can refer suspected/confirmed cases of oral cancer, few report maintaining a record of these oral cancer cases which could improve the relationship of PHC with specialized care. Early diagnosis of oral cancer increases the chances of treatment success. Thus, this situation has great ethical and legal implications, considering that late diagnosis and management results in a poor outcome, including a failure to follow-up diagnosed patients. The importance of such diagnoses in PHC has been discussed, as well as professionals’ failure to identify lesions in the initial phase. Therefore, it is important to emphasize its importance to professionals and to encourage continued professional development for its management. In addition, as already occurs in some OHTs, campaigns for oral examination should be encouraged [6,9,13,14].

North and north-east regions are the less developed Brazilian regions [2,9]. Furthermore, data from the last Brazilian Dental Epidemiological Survey (SB Brazil 2010) show that the oral health status of Brazilians of the northern and north-eastern regions are the worst, especially regarding dental caries, periodontal diseases and the need for dental prostheses [15]. Moreover, it is possible to observe three Brazilian realities, in which the north-east and mid-west perform similarly, the southern and south-eastern regions have a higher rate of dental procedures, and the north lower. Furthermore the north-eastern region has a higher number of OHTs, it performs, on average, a smaller number of procedures when compared with the southern and south-eastern regions. The difference in the rate of procedures performed in each Brazilian geographic region emphasizes the great differences in sociodemographic and health conditions of Brazil, and is widening internal inequalities. The differences in the rate of dental procedures could be explained by the fact that the access of people in deprived regions is more difficult and also due to the differences in regulation of OHTs. Another explanation for the differences observed among the Brazilian regions could be that a high proportion of OHTs in the north and north-east have only recently been established. More empirical studies should be carried out for a better understanding of these differences.

Although the present study results are limited to the use of a secondary database, where the performance of dental procedures is based on the report of the dentist, there is evidence of the need to expand the supply of prostheses and the early diagnosis of oral cancer in PHC. The evaluation of the primary dental health-care service makes it possible to outline strategies for the strengthening of access to oral health care for the Brazilian population. Thus, considering that the SUS is a universal health system that aims to serve all individuals of all age groups, it is necessary to reorganize the service in order to increase the variety of procedures offered according to the needs of the population, with the aim of achieving integrated oral health care.

## 5. Conclusions

Brazilian regions with the highest volume of dental need deliver the lowest number of dental procedures. The need to tackle inequalities and further shape the supply of appropriate primary health care (PHC) is evident.

## Figures and Tables

**Table 1:** Basic dental procedures performed by oral health teams, Brazil, 2013–2014.

Variables	Yes (%)
Preventive procedures	
Supragingival scaling, root planing and coronal polishing	17,551 (96.9)
Fluoride application	17,866 (98.6)
Does OHT guarantee an appointment scheduling for the continuity of treatment?	16,639 (91.9)
Restorative/Prosthetic procedures	
Anatomical and functional impression for prostheses	1478 (8.2)
Return visit to evaluate prostheses installation	2231 (12.3)
Prostheses cementation	5109 (28.2)
Does the OHTs promote actions in its territory to identify people who need dental prostheses?	9504 (52.5)
Amalgam filling	16,180 (89.3)
Composite filling	17,718 (97.8)
Deciduous tooth restoration	17,851 (98.5)
Surgical procedures	
Removal of cysts	3883 (21.4)
Frenectomy	4929 (27.2)
Removal of impacted teeth	5446 (30.1)
Ulotomy/ulectomy	11,915 (65.8)
Suture of trauma injuries	14,485 (80.0)
Drainage of oral abscesses	15,908 (87.8)
Alveolitis treatment	16,332 (90.2)
Permanent tooth extraction	17,746 (98.0)
Deciduous tooth extraction	17,842 (98.5)
Endodontic procedures	
Pulpotomy	15,093 (83.3)
Access to dental pulp	15,959 (88.1)
Cancer monitoring	
Does OHT have documents proving the registration of suspected/confirmed cases of oral cancer?	4128 (22.8)
Does OHT have reference to forwarding suspected/confirmed cases of oral cancer?	14,497 (80.0)

Note. OHT = Oral Health Team.

**Table 2:** Average number of dental procedures performed by oral health teams in Brazilian geographical regions, 2013–2014.

Brazilian Geographical Regions (Human Development Index) *	Population Size	Number of OHTs	Dental Procedures			
			Mean	SD ^†^	Min ^‡^	Max ^§^
North (0.667)	15,864,454	1263	13.9	2.9	0	21
North-east (0.663)	53,081,950	7700	14.9	2.8	0	23
Mid-west (0.757)	14,058,094	1572	15.1	2.7	0	23
South-east (0.766)	80,364,410	5027	16.5	2.5	0	23
South (0.754)	27,386,891	2552	16.5	2.4	2	23
Brazil	190,755,799	18,114	15.5	2.8	0	23

Note. * In accordance with the United Nations Development Programme [8] the Human Development Index (HDI) summarizes the measure of life expectancy at birth, mean of years of schooling for adults aged 25 years and over, and gross income per capita. The scores varied from 0 to 1 (the higher the score the better the outcome); ^†^ SD = Standard Deviation; ^‡^ Min = Minimum values; ^§^ Max = Maximum values.
